# FGF receptors are required for proper axonal branch targeting in *Drosophila*

**DOI:** 10.1186/s13041-019-0503-y

**Published:** 2019-10-24

**Authors:** Júnia Vieira dos Santos, Renee Yin Yu, Andrea Terceros, Brian Edwin Chen

**Affiliations:** 1 Centre for Research in Neuroscience, Research Institute of the McGill University Health Centre, Montréal, Québec, Canada; 2 Departments of Medicine, Neurology and Neurosurgery, McGill University, Montréal, Québec, Canada

## Abstract

Proper axonal branch growth and targeting are essential for establishing a hard-wired neural circuit. Here, we examined the role of Fibroblast Growth Factor Receptors (FGFRs) in axonal arbor development using loss of function and overexpression genetic analyses within single neurons. We used the invariant synaptic connectivity patterns of *Drosophila* mechanosensory neurons with their innate cleaning reflex responses as readouts for errors in synaptic targeting and circuit function. FGFR loss of function resulted in a decrease in axonal branch number and lengths, and overexpression of FGFRs resulted in ectopic branches and increased lengths. FGFR mutants produced stereotyped axonal targeting errors. Both loss of function and overexpression of FGFRs within the mechanosensory neuron decreased the animal’s frequency of response to mechanosensory stimulation. Our results indicate that FGFRs promote axonal branch growth and proper branch targeting. Disrupting FGFRs results in miswiring and impaired neural circuit function.

## Introduction

Molecular cues allow for axon guidance, target recognition, and synaptogenesis, and along with activity-dependent plasticity, lead to the formation of functioning neural circuits [1]. One molecular strategy used for neuronal wiring is to promote proliferation of neurons that reach their appropriate target using neurotrophic factors [2]. These factors can also induce axonal growth and regulate neuronal survival [3]. Previous studies have demonstrated the strong axonal growth induction potential of neurotrophic factors such as Fibroblast Growth Factor (FGF) [4, 5] and Nerve Growth Factor (NGF) in cultured cells [6, 7] and animal models [8–10]. However, it is not clear how they might mediate specific axonal targeting decisions that determine neuronal connectivity.

Fibroblast Growth Factors (FGFs) are polypeptides that can be secreted to promote paracrine signaling [11] or act intracellularly as modulators of voltage-gated channels [12]. FGFs function in a wide variety of developmental processes ranging from axis patterning in embryos [13] to induction and maintenance of cell lineages [14]. In humans, the functional diversity of FGFs arises from the combinations of 22 FGFs and four FGF receptors (FGFRs) [15]. In *Drosophila melanogaster*, there are three FGF ligands, *Branchless* (*Bnl*), *Pyramus* (*Pyr*) and *Thisbe* (*Ths)* [16, 17], and two FGFRs, *Heartless* (*Htl*) and *Breathless* (*Btl*) [18]. Binding of FGF to its receptor promotes dimerization of FGFRs, initiating a molecular cascade that activates phosphatidylinositol 3-kinase-Protein Kinase B (PIP3-Akt) and Ras-mitogen-activated protein kinase (MAPK) signaling pathways [19, 20]. The FGFR signaling pathways are highly conserved in metazoan organisms [21], indicating that FGF and FGFR signaling might function similarly in the neural development of different species.

Vertebrate FGF family members serve a variety of functions throughout neural and synapse development [22]. Vertebrate FGF1 and FGF2 promote neurite outgrowth, whereas FGF2 also contributes to survival of cultured chick neurons [23]. Other FGFs such as FGF7, FGF10, and FGF22, play a role not only in neurite outgrowth but also in presynaptic differentiation [24]. However, it is not known whether FGFs contribute to the specificity of neural wiring. Here we examine FGFR contributions to hard-wired neural circuit formation using the mechanosensory system of *Drosophila melanogaster* [25, 26]. We find that loss of function of FGFRs in single mechanosensory neurons results in disruptions of synaptic connectivity, including loss of axonal branches. Overexpression of the two *Drosophila* FGFRs results in targeting errors of ectopic branching. These impairments in mechanosensory wiring reduced the animal’s ability to respond to mechanical stimuli and elicit a hard-wired cleaning response.

## Material and methods

### Fly stocks

The following fly stocks were obtained from the Bloomington Drosophila Stock Center: P{w[+m*] = nSyb-GAL4.S}3, *P{w[+mC] = UAS-btl.lambda}2*, *P{w[+mC] = UAS-btl.lambda}1, P{w[+mC] = UAS-btl::GFP-S65 T}3*, *P{y[+t7.7] v[+t1.8] = TRiP.HMS02038}attP2, P{y[+t7.7] v[+t1.8] = TRiP.HMS02656}attP40*, *P{y[+t7.7] v[+t1.8] = TRiP.HMC04140}attP2*, *P{y[+t7.7] v[+t1.8] = TRiP.HMS05005}attP40*, *P{w[+mC] = GAL4-btl.S-hsFLP}2*, *P{w[+mC] = UASp-Act5C.T:GFP}2; btl[724] P{w[+mW.hs] = FRT(w[hs])}2A P{ry[+t7.2] = neoFRT}82B / TM3, P{w[+mC] = tubP-GAL80}3, P{w[+mC] = UAS-htl.M}YYDFR-F16*, *P{y[+t7.7] v[+t1.8] = TRiP.HMS01437}attP2*, *P{y[+t7.7] v[+t1.8] = TRiP.HMJ22375}attP40*, *P{y[+t7.7] v[+t1.8] = TRiP.HMS04514}attP40*, *P{y[+t7.7] v[+t1.8] = TRiP.HMS01046}attP2*, *P{y[+t7.7] v[+t1.8] = TRiP.HMJ30113}attP40*, *P{w[+mC] = UAS-htl.DN.M}33-B40; P{w[+mC] = UAS-htl.DN.M}33-B61.* The *455-Gal4* line was crossed with *UAS-dsRNA Btl*, *UAS-dsRNA Bnl, UAS-dsRNA Htl* or *UAS-dsRNA Pyr* to induce RNA interference (RNAi) solely within the scutellum. To overexpress *Btl* and *Htl*, we used *455-Gal4* to selectively drive UAS expression. The *Btl* rescue line was obtained by crossing *UAS-Btl* to the respective MARCM *Btl*^*null*^ line while *Htl* rescue was obtained by crossing *455-Gal4 / CyO; UAS-Htl / TM6b* to *Htl* *DN*. *DC1.4-Gal4* (a kind gift from Dr. Pat Simpson) was used to selectively express *UAS-dsRNA Btl* or *UAS-Btl* in the DorsoCentral neurons [27]. *Repo-Gal4* was used to drive UAS expression selectively in glial cells. MARCM *Btl*^*null*^ and *Btl* rescue lines were heat-shocked at 37 °C for 1 hour at the pupal stage.

### Fluorescence in situ hybridization (FISH)

Fluorescence in situ hybridization for *Btl* mRNA or *Htl* mRNA was performed as described previously [28]. Female adult n*Syb-Gal4; UAS-mCD8-GFP* fly thoraxes were collected and fixed in 4% formaldehyde in PBS for 30 min at room temperature on a shaker, then transferred to cold Dent’s fixative (80% Methanol, 20% DMSO) and stored at − 20 °C overnight. The samples were then washed in 100 mM Tris/HCl (pH 7.4), 100 mM NaCl for 1 h at room temperature on a shaker, then transferred to 15% fish gelatin/15% sucrose for incubation at room temperature overnight on a shaker. Samples were then transferred to 25% fish gelatin/15% sucrose and incubated at room temperature overnight on a shaker. Finally, the samples were positioned on their sides in plastic molds containing 20% fish gelatin/15% sucrose, flash-frozen with dry ice and stored at − 80 °C. The samples were cut at 10 μm thick along the rostral-caudal, dorsal-ventral axis, mounted on slides and dried. Sample outlines were traced with a water repellent pen on the slide. The sections were first re-permeabilized with 70% ethanol for 10 min. Forty-eight custom Stellaris® FISH Probes conjugated to Quasar 570 were designed against *Btl* or *Htl* mRNA sequence by utilizing the Stellaris® RNA FISH Probe Designer version 4.2 (Biosearch Technologies, Inc., Petaluma, CA), available online at www.biosearchtech.com/stellarisdesigner. Probe mix was made by adding the probes to hybridization buffer at a concentration of 125 nM, and the probe mix was dispensed onto the slide. The slide was placed in a humidified chamber and incubated for 12 h overnight in the dark at 37 °C. The probe mix was removed, wash buffer was dispensed on the slide, and incubated in the dark for 30 min at 37 °C. Two more washes with wash buffer were performed. Finally, the slide was stained and mounted with antifade mountant with DAPI. Fluorescence microscopy was performed using an Olympus laser-scanning confocal microscope FV1000. Images were acquired using a 40× (N.A. 1.3) or 60× oil objective (N.A. 1.4).

### Carbocyanine dye labeling

As described previously [26], the morphology of *Drosophila* pSc mechanosensory axons was visualized via lipophilic carbocyanine dye injection into the open socket of the pSc bristle. We plucked the bristles of two-day old female flies and left them overnight in fixative solution (Paraformaldehyde, 3.7%, in 0.2 M carbonate-bicarbonate buffer at pH 9.5). The left and right pSc neurons were labelled using fluorescent carbocyanine dyes, DiI and DiD (Invitrogen™, ThermoFisher, Inc.) dissolved in ethanol (final concentration: 20 mg/mL and 40 mg/mL, respectively). The dye-filled flies were left undisturbed and protected from light in a Petri dish, partially immersed in 0.2 M carbonate-bicarbonate buffer at pH 9.5. After forty-eight hours, the thoracic ganglion was dissected and placed on a slide and imaged under a coverslip.

### Image acquisition and analysis

Fluorescent images were acquired using a Zeiss AxioScope A1 epifluorescence microscope or an Olympus laser-scanning confocal microscope FV1000, with a 40× objective (N.A. 1.3). Transmitted light images were acquired and used to evaluate dissection quality and to measure thoracic ganglia width. Only images without CNS damage or surface occlusions were included for data analysis. Maximal intensity projections were stacked from optical sections and adjusted for contrast and brightness if required. Measurements of axonal branch length and branch number were performed blind to genotype. Custom-written software in MATLAB was used to perform the quantitative image analysis.

### Behavioral assay and analysis

The behavioral assay and analysis was performed as previously described [26]. The *455-Gal4* driver was used to selectively express *UAS-dsRNA* in the scutellum to ensure that RNA interference did not perturb the postsynaptic neural circuitry. Two-day old female flies were decapitated and placed in a humidified chamber to recover over a period of 1 hour. After recovery, flies standing on their six legs were tested for responsiveness to stimulation of the anterior notopleural bristle (aNp) with forceps to elicit a cleaning reflex from the anterior legs. Only responsive flies were used for further evaluation by pressure injection of fluorescent dye onto the posterior scutellar bristles. The presence or absence of response was scored visually, with a positive response scored as the successful movement of the rear pair of legs towards the labelled bristles and spread of the dye to the rear legs.

### Experimental design and statistical analysis

In order to reduce the variability due to environmental conditions, age, and sex, flies were reared at 25 °C on standard cornmeal under 12 h light/dark cycles and experiments were performed on 2-day old adult female animals. Preliminary unblind experiments were conducted on 10 animals for dye labeling experiments and 30 animals for behavioral assays per genotype to determine adequate sample size through power analysis.

All statistical analysis was performed using SPSS (version 25) and GraphPad Prism (version 7.04). A total of 19 wildtype, 32 *Btl RNAi*, 10 *Btl*^*null*^, 34 *Btl* overexpression, 19 *Btl* rescue, 20 *Bnl RNAi*, 14 *Pyr RNAi*, 38 *Htl RNAi,* 11 *Htl DN*, 21 *Htl* overexpression and 10 *Htl* rescue mutant neurons were analyzed. Shapiro-Wilks test was performed to assess normality on the dataset, and all groups except *Btl* rescue and *Htl* overexpression had a *p* value higher than 0.05, confirming that the datasets came from Gaussian distributions. One-way ANOVA followed by Dunnett’s post hoc pairwise comparison was performed to assess statistical significance in axonal branch length between wildtype and mutant phenotypes. For *Btl* rescue and *Htl* overexpression, Mann Whitney *U* test was performed to assess statistical significance in axonal branch length between wildtype and mutant phenotypes. Kruskal-Wallis non-parametric *H* test followed by Dunnet’s post hoc comparison was performed to assess statistical significance in number of branches between wildtype and mutant phenotypes for the above mentioned groups. Qualitative analysis of axonal variability was performed blind to genotype by randomly distributing the entire axonal arbor dataset. A total of 35 phenotypes were identified among wildtype and mutant axons. These phenotypes were further subdivided into 10 non-mutually exclusive base phenotypes, used for calculation of frequency of targeting errors. Twenty axonal branching patterns were only present in mutant phenotypes (7 *Btl RNAi*, 9 *Btl* overexpression, 9 *Htl RNAi* and 5 *Htl* overexpression). Quantification of axonal branches and lengths for all genotypes are shown in Additional file 1: Table S1. For the behavioral assay, 116 wildtypes, 113 *Btl RNAi*, 125 *Btl* overexpression, 107 *Htl RNAi* and 76 *Htl* overexpression flies were tested. To assess association between the wildtype and mutants in the behavioral assay, a *χ2* test for independence was performed, and all groups had a *p* value higher than 0.05, indicating no significant association between the wildtype and mutants. A two-tailed *t* test for proportions was performed to assess statistical significance in the frequency of response between control and each genotype.

## Results

### FGF receptors are expressed in *Drosophila* mechanosensory neurons

**Fig. 1:**
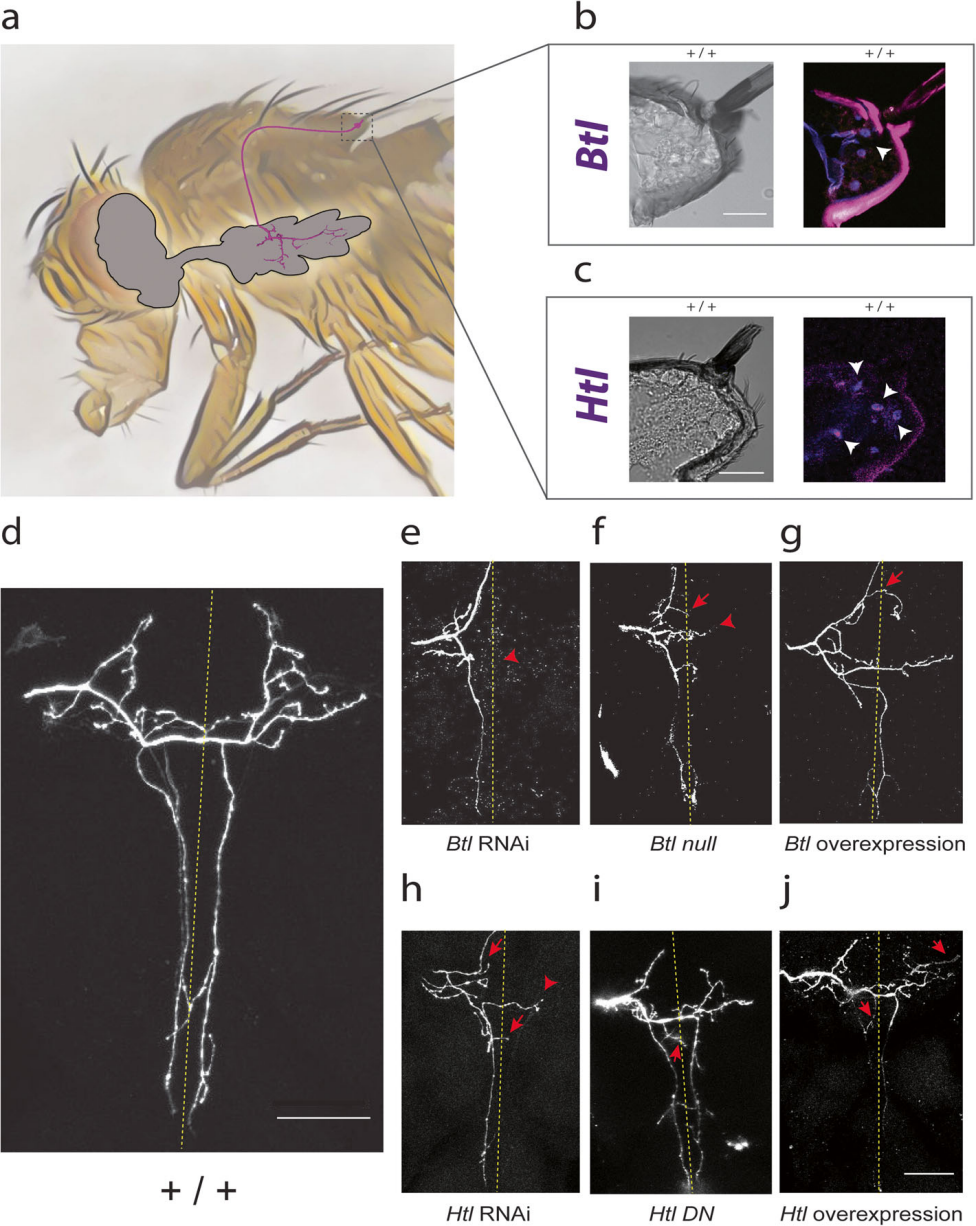
– The FGFRs *Btl* and *Htl* are required for proper synaptic connectivity. **a** The *Drosophila* mechanosensory neurons innervate the bristles of the notum. Each bristle is innervated by a single mechanosensory neuron which is uniquely identifiable based on its location. The right pSc neuron and its axonal arbor within the thoracic ganglion are illustrated in magenta. **b** A cross section of a pSc bristle is shown in brightfield and the corresponding *Btl* fluorescence in situ hybridization signal in magenta. *Btl* mRNA (arrowhead) was detected in the neuron underlying the pSc bristle. **c** A cross section of a pSc bristle is shown with *Htl* fluorescence in situ hybridization signal in magenta (arrowheads). *Htl* mRNA was detected in the pSc neuron and its surrounding cells. **d** The pSc mechanosensory neuron has a unique and stereotyped axonal branching pattern within the central nervous system. The stereotyped connectivity of the pSc neuron can be used to identify axonal targeting errors in mutants. **e**-**g** Mis-regulation of *Btl* expression results in synaptic targeting errors. **e** Knockdown of *Btl* using RNAi resulted in a loss of core branches. **f** *Btl*^*null*^ MARCM mutants also had missing core branches (arrowhead), mostly contralateral to the axonal entry site into the thoracic ganglion. **g** Increasing *Btl* expression resulted in misrouting errors as well as ectopic branch formations. **h**-**j** Mis-regulation of *Htl* expression also results in targeting errors. **h** Knockdown of *Htl* using RNAi resulted in ectopic branches (arrows) and loss of secondary branches (arrowhead). **i** *Htl* Dominant Negative (*Htl* *DN*) mutants showed similar axonal targeting errors as *Htl* RNAi mutants such as ectopic branches towards the midline (arrow). **j** Overexpression of *Htl* also resulted in ectopic branches (arrows). Scale bars, 20 μm in (**b**, **c**), 50 μm in (**d**, **j**). Arrowheads point to absent branches, arrows point to ectopic branches

We use the *Drosophila* mechanosensory system to examine hard-wired synaptic targeting. In this system, each large bristle on the fly notum is innervated by a single mechanosensory neuron whose axon projects within the thoracic ganglion to form specific synaptic connections (Fig. 1). Each mechanosensory axonal arbor is identifiable by its unique and invariant branching pattern. Here we focused our experiments on the left and right posterior scutellar (pSc) neurons due to its accessible anatomical location on the posterior scutellum (Fig. 1a) and its genetic accessibility using the *455-Gal4* driver. First, we sought to determine whether the *Btl* or *Htl* FGFRs were transcribed in mechanosensory neurons. We used fluorescent in situ hybridization for detection of *Btl* or *Htl* mRNA and identified their expression within pSc neurons (Fig. 1b, c). Interestingly, *Btl* mRNA was consistently identified in pSc neurons (*n* = 7) and absent in other cell types such as the neighboring glial cell, whereas *Htl* mRNA (*n* = 4) exhibited a more widespread expression pattern, including within the supporting cells (Fig. 1b, c).

### FGFRs are necessary for proper synaptic targeting

To examine the role of FGFRs in synaptic targeting, we used the invariant axonal branching pattern of pSc neurons as a readout for axonal targeting errors (Fig. 1d) [28–31]. First, to quantify the baseline variability in wildtype synaptic targeting, we characterized the pSc axonal arbor by measuring the total number of branches and branch lengths in 19 control *455-Gal4*/+ animals. A schematic of the control axonal arbor was generated based on the frequency of branch occurrence (Additional file 1: Figure S1), and is consistent with our previous measurements of pSc neurons in wildtype animals [28, 29] and *455-Gal4* animals [30, 31].

To examine how *Btl* is used for pSc axonal targeting, we first expressed *dsRNA* for *Btl* solely within the pSc neuron using *455-Gal4* driving *UAS-dsRNA Btl* to induce RNA interference. RNAi knockdown of *Btl* resulted in disruptions of the pSc axonal branching pattern with noticeable reductions in the number of secondary and tertiary axonal branches (Fig. 1**e**). To confirm these observations using a genetic knockout of *Btl*^*null*^ within pSc axons, we used the Mosaic Analysis with a Repressible Cell Marker (MARCM) technique to produce homozygous *Btl* loss of function within only a few cells in the mosaic animal (Fig. 1**f**). We found that the homozygous *Btl*^*null*^ pSc axons in these MARCM flies phenocopied the RNAi knockdown of *Btl* (*n* = 10 and 32, respectively), displaying a noticeable decrease in branching complexity and loss of branches. Whereas *Btl* knockdown and loss of function axonal arbors displayed a reduction of secondary and tertiary branches, overexpression of *Btl* within pSc neurons (*455-Gal4 / UAS-Btl*, *n* = 34) resulted in a pronounced increase in the number of ectopic branches (Fig. 1g). Importantly, none of these experimental manipulations altered the axon guidance of the pSc axon from the periphery into the central nervous system via the mesothoracic root.

Is the axonal branch growth-promoting function of *Btl* shared with the other FGFR *Htl*? We evaluated the axonal arbors of pSc neurons in different *Htl* mutant animals (Fig. 1h**-**j). *Htl* RNAi mutants (*455-Gal4 / UAS-dsRNA Htl*) showed an increased frequency of ectopic branches anteriorly while they also had a significant reduction of secondary and tertiary branches (Fig. 1h). To verify these RNAi results, we analyzed the axonal arbors of pSc neurons overexpressing *Htl* Dominant Negative, which disrupts Htl protein function (*455-Gal4*; *UAS-Htl DN*) (Fig. 1i). We observed that *Htl DN* mutants displayed a partial phenocopy of *Htl* RNAi targeting errors (*n* = 11 and 38, respectively), including the presence of ectopic branches in anterior region of the thoracic ganglion. Similar to *Btl* overexpression mutants, *Htl* overexpression mutants showed an increase in the number of ectopic branches (Fig. 1j).

**Fig. 2:**
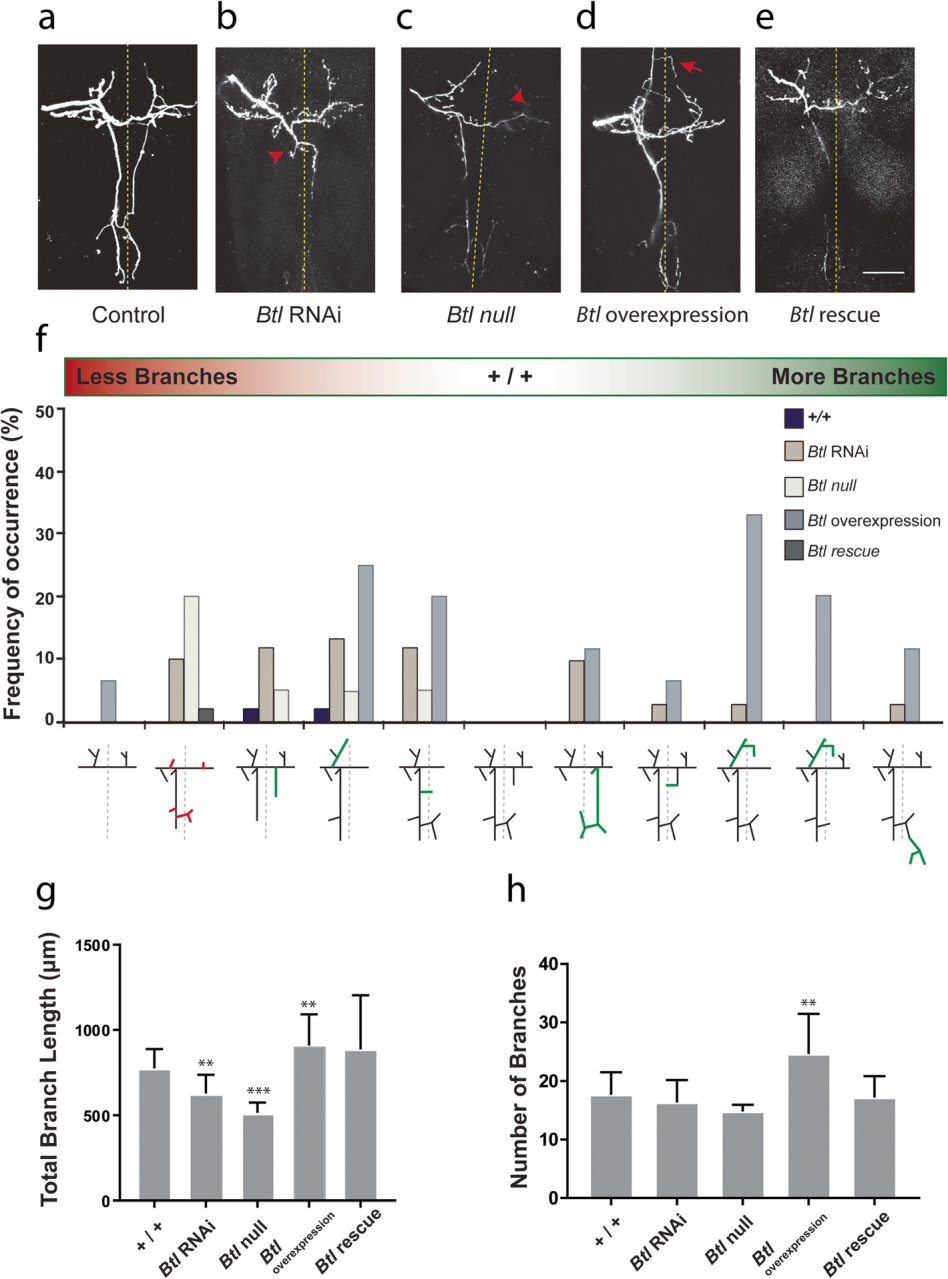
- Altered *Btl* expression results in axonal branch growth errors and misrouting. **a**-**d** Disrupted *Btl* expression produced a higher frequency of axonal branching errors. Expression of *Btl* dsRNA solely within the pSc neuron had a higher frequency of routing errors and absence of core branches (**b**), similar to *Btl*^*null*^ MARCM mutants (**c**). *Btl* RNAi and *Btl*^*null*^ mutants both showed significant decreases in total branch lengths. **d** *Btl* overexpression resulted in an increase in total branch length and significantly more ectopic axonal branches and routing errors. **e** Rescue expression of *Btl* in MARCM mutants reverted the axonal branching patterns to wildtype. Red indicates reduction of branches, green indicates ectopic branches. **f*** Btl* loss of function versus overexpression had opposing effects on axonal branch length (**g**) and number of branches (**h**). Scale bar, 50 μm. Arrowheads point to absent branches, arrows point to ectopic branches. Error bars are S.D.

Quantitative analysis showed a significant difference (*F* (3.92) = 4.732, *p* = 0.0041) in total axonal branch lengths between control, *Btl* RNAi, *Btl*^*null*^ MARCM and *Btl* overexpression (Fig. 2). Compared to control animals (Fig. 2a), *Btl* RNAi and *Btl*^*null*^ (Fig. 2b, c, respectively) neurons showed a significant decrease (*n* = 32, *p* = 0.0067 and *n* = 10, *p* < 0.0001, respectively) in total axonal branch lengths. Conversely, *Btl* overexpression (Fig. 2d) resulted in a significant increase (*n* = 34, *p* = 0.0031) in total branch length compared to wildtype. In *Btl* RNAi and *Btl*^*null*^, the decrease in arbor complexity was mostly due to decreased lengths of secondary and tertiary branches rather than arbors with less branches (Fig. 2f–h). Axonal arbors with ectopic branches were more frequently observed in *Btl* overexpression, resulting in a significant increase in the total number of branches (*p* = 0.0057) compared to controls (Fig. 2g). For *Btl* rescue experiments (*n* = 19) (Fig. 2e), we observed no significant differences in total branch length or branch number compared to controls.

**Fig. 3:**
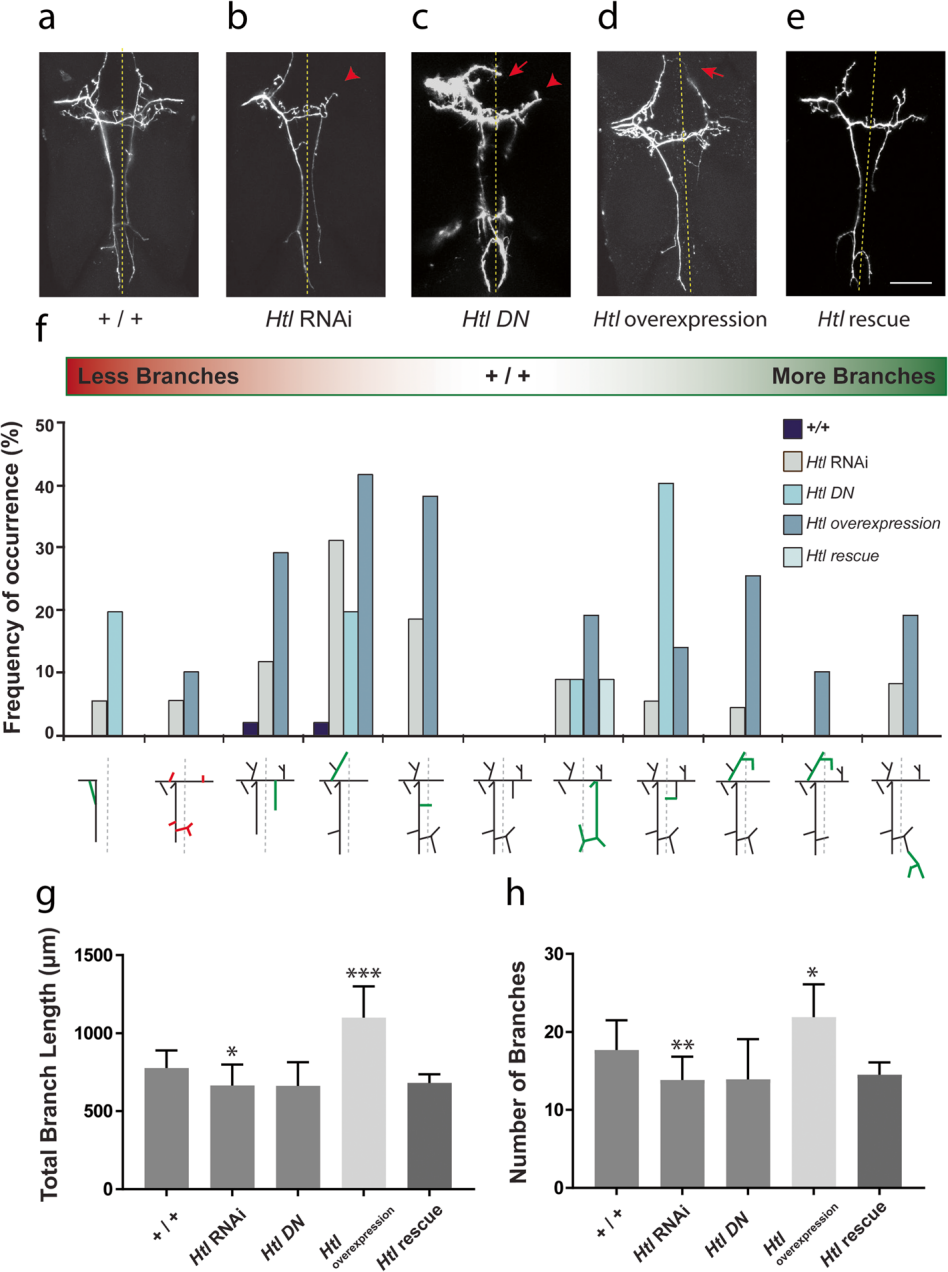
– Altered expression of *Htl* disrupts pSc neuron synaptic connectivity. **a**-**d** *Htl* mutants have a higher frequency of synaptic targeting errors. Representative examples of wildtype pSc axonal arbors (**a**), *Htl* RNAi (**b**), *Htl* Dominant Negative (**c**) and *Htl* overexpression (**d**). **e** Overexpression of *Htl* with the *Htl DN* in the pSc neuron reverted the axonal branching pattern to wildtype. **f** *Htl* mutants had stereotyped pSc axonal targeting errors, similar to *Btl* mutants. Red indicates reduction of branches, green indicates ectopic branches. Quantitative analysis showed a significant decrease in the total branch length (**g**) and total number of branches (**h**) in *Htl* RNAi compared to control arbors. In contrast, *Htl* overexpression within the pSc neuron resulted in a higher occurrence of axonal branch misrouting and ectopic branches, but also missing core branches (**f**). Overall, *Htl* overexpression resulted in a significant increase in total branch length (**g**) and total number of branches (**h**) compared to control. Scale bar is 50 μm. Arrowheads point to absent branches, arrows point to ectopic branches. Error bars are S.D.

Statistical analysis of *Htl* mutants showed a significant difference (*F* (3.73) = 3.154, *p* = 0.0299) in total axonal branch lengths between controls, *Htl* RNAi, *Htl DN* and *Htl* rescue (Fig. 3). Neurons expressing *Htl* RNAi (Fig. 3b) showed a significant decrease (*n* = 38, *p* = 0.0161) in total axonal branch lengths, whereas overexpression of *Htl* (Fig. 3d) resulted in a significant increase in total branch length (*n* = 21, *p* < 0.0001) (Fig. 3f–h). *Htl* RNAi mutants also had significantly fewer secondary and tertiary core branches (*p* = 0.0046) in addition to reduced branch lengths, resulting in an overall reduction in axonal arbor complexity compared to control neurons (Fig. 3f–h). Overexpression of *Htl* in pSc axons resulted in a significant increase in total number of branches (*n* = 21, *p* = 0.0185, Fig. 3d) due to a large number of ectopic branch formations. For *Htl DN* (*n* = 11) and *Htl* rescue (*n* = 10) experiments (Fig. 3c and e, respectively), there were no significant differences in total branch length or branch number.

Both FGFRs *Btl* and *Htl* were required for proper branch routing as well. Loss of function and overexpression mutants for both genes displayed stereotyped errors related to inappropriate axonal branch routing. Midline crossing errors were more frequently observed in overexpression mutants (representative examples in Figs. 1g, 2d, 3d), while branch misrouting was equally observed among knockdown and overexpression mutants (Fig. 2b). Frequency of midline crossing errors and ectopic branches for all genotypes are shown in Additional file 1: Table S1.

### Disruption of *Btl* and *Htl* expression in the pSc axon impairs the cleaning reflex

**Fig. 4:**
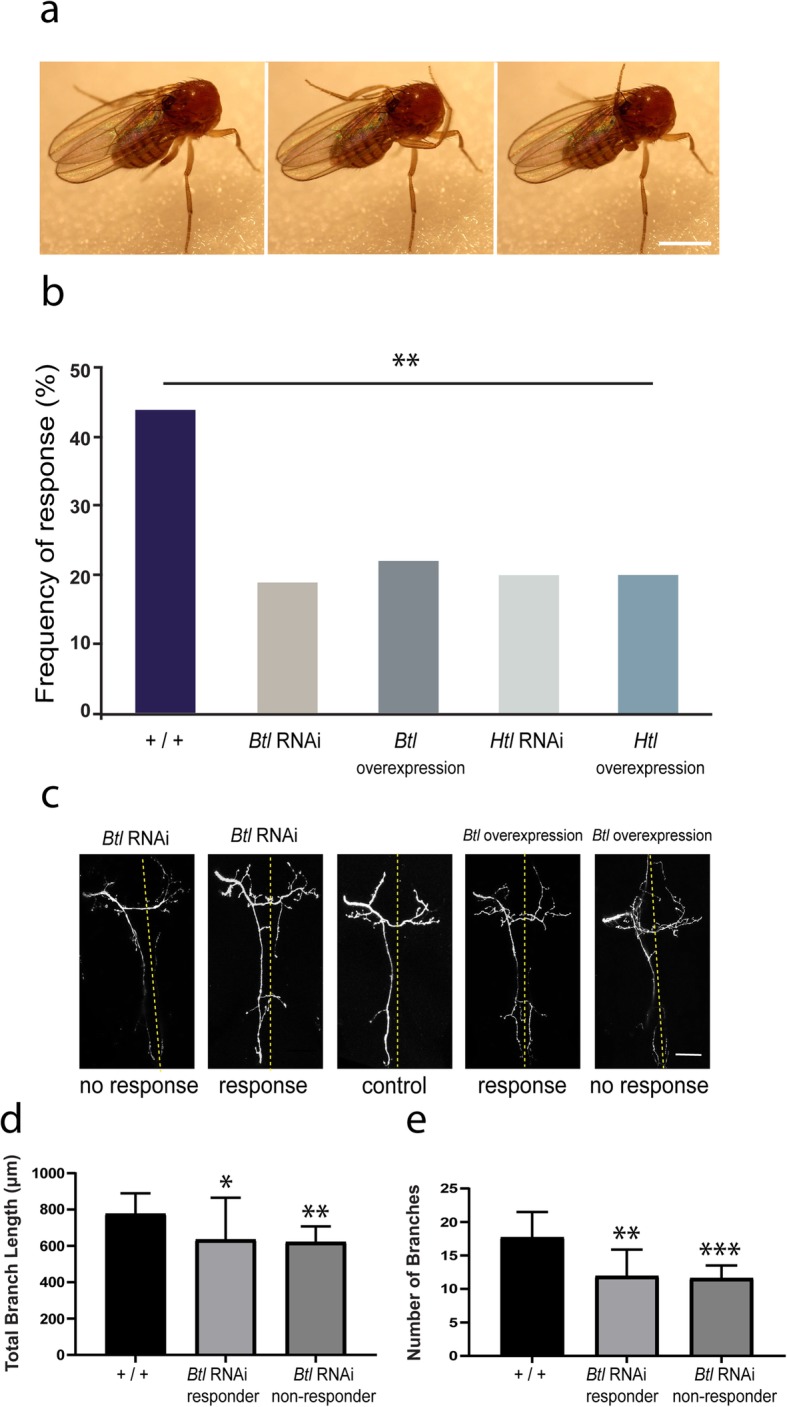
– Axonal targeting errors impair neuronal connectivity and mechanosensation. **a** Stimulation of the pSc bristle via application of fluorescent dye triggers a grooming response. A positive response was visually scored as the successful movement of the rear pair of legs onto the labelled bristles with spread of the dye to the rear legs. Scale bar is 1 mm. **b**, **c** Disrupting the pSc connectivity led to a significant decrease (*p* < 0.01) in response rate of all *Btl* and *Htl* mutant groups when compared to control. **c** Direct correlation of the pSc axonal branching pattern with the animal’s behavioral response revealed that animals with more severe axonal targeting errors (“no response”, far left and far right images) were more likely to be non-responsive to mechanical stimulation. Scale bar is 50 μm. Total branch lengths (**d**) and branch numbers (**e**) were significantly smaller in *Btl* RNAi responders (*p* < 0.05) and *Btl* RNAi non-responders (*p* < 0.01) compared to controls. Error bars are S.D.

We next investigated whether the observed disruptions in pSc synaptic connectivity affected neural circuit function by testing the behavioral response of FGFR mutant mosaic animals. In wildtype animals, mechanical stimulation of the pSc bristle evokes a cleaning reflex, where the third pair of legs grooms the bristle in an attempt to end the stimulus (Fig. 4a). We used the *455-Gal4* driver to knock down or overexpress *Btl* and *Htl* within the pSc neuron without affecting the downstream mechanosensory circuitry. We found that all mutant groups displayed a significantly reduced response rate (*p* < 0.01) in the cleaning reflex when compared to controls (Fig. 4b).

To determine if the presence of targeting errors would lead to a lower response rate, we analyzed the axonal arbors of animals with a positive grooming reflex (responders) and animals without a grooming reflex response (non-responders) in *Btl* RNAi mutants (Fig. 4c). Quantitative analysis revealed a significant difference (*F* (2,42) = 4.158, *p* = 0.0052) between the control animals, *Btl* RNAi responders and *Btl* RNAi non-responders. *Btl* RNAi responders and non-responders showed a significant decrease (*n* = 13, *p* = 0.0062 and *n* = 10, *p* = 0.0244, respectively) in total branch lengths (Fig. 4d) and branch numbers (*p* < 0.0001 and *p* = 0.0036, respectively) (Fig. 4e) compared to controls.

### Knockdown of FGFs, *Bnl* and *Pyr*, partially phenocopies FGFR mutants

**Fig. 5:**
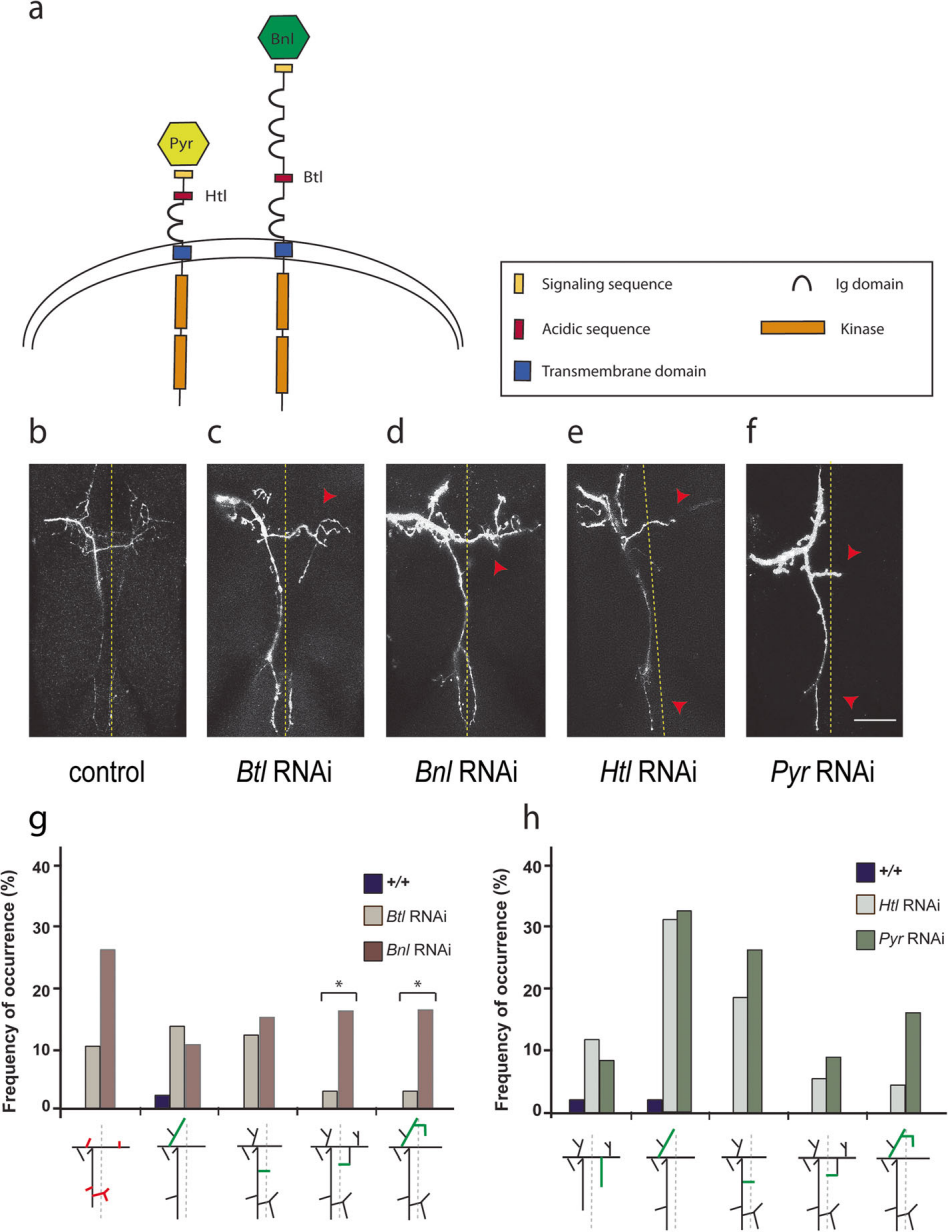
– Specific axonal targeting errors are phenocopied in FGF ligand and FGFR mutants. **a** The FGF ligands Bnl and Pyr bind to their respective FGFRs Btl and Htl at the cell surface. **b**-**f** Representative examples of a wildtype axonal arbor (**b**), *Btl* RNAi (**c**), *Bnl* RNAi (**d**), *Htl* RNAi (**e**) and *Pyr* RNAi (**f**) are shown. **g** Five error types had significantly greater frequency of occurrence in both *Btl* RNAi and *Bnl* RNAi mutants compared to wildtype flies. Two targeting error phenotypes had a significantly higher frequency of occurrence (*p* < 0.05) in the *Bnl* RNAi mutants than in *Btl* RNAi mutants. **h ***Pyr* RNAi mutants also had five error types that occurred at significantly greater frequency than in wildtype flies that were also found in *Htl* RNAi mutants. Scale bar is 50 μm. Arrowheads point to absent branches, arrows point to ectopic branches

To verify the signaling mechanism by which the FGFRs control axonal branch growth, we performed RNAi knockdown experiments on the FGFs *Bnl* and *Pyr*, the ligands for *Btl* and *Htl*, respectively. Because FGF/FGFR signaling can act in an autocrine and paracrine manner and it is not known which cells might express the FGF ligands, we used the *455-Gal4* driver to express the dsRNA for *Bnl* and *Pyr* in the primordial scutellum (Fig. 5). *455-Gal4* is active in the larval imaginal discs and persists throughout pupal development, being expressed in all cells that give rise to the scutellum, including the epithelial cells and sensory organ precursors that will become the pSc neuron, pSc glial, pSc socket cell, and other surrounding cells that create the pSc bristle structure. We found that the axonal arbors of *455-Gal4 / UAS-dsRNA Bnl* animals had five axonal targeting error patterns that were also present in *Btl* RNAi mutants (Fig. 5c, d), of which the frequency of occurrence for two targeting error types were significantly higher (*p* = 0.0466) in *Bnl* RNAi mutants (*n* = 20) than *Btl* RNAi mutants (*n* = 32) (Fig. 5g). In *Pyr* RNAi mutants, we also observed five targeting error types that were also found in *Htl* RNAi mutants (Fig. 5e, f). However, the frequency of occurrence of these phenocopies did not differ significantly between *Pyr* RNAi and *Htl* RNAi mutants (Fig. 5h).

## Discussion

In insects, FGFRs modulate gene expression, cellular interactions, and cellular morphology [13, 20, 32]. During *Drosophila* organogenesis, FGF ligands promote dorsal migration of ectoderm and radial movement of neuroectoderm [16, 33, 34]. The *Htl* FGFR regulates cellular migration within the mesoderm during embryonic development [35]. The *Btl* FGFR plays a central role in the development of the *Drosophila* respiratory system by inducing tracheal tube branching [36, 37]. Our results add a novel function to *Drosophila* FGFRs in axonal arbor formation. We found that downregulation of *Btl* or *Htl* expression resulted in loss of axonal arbor size and number of branches, whereas overexpression of FGFRs resulted in more frequent routing errors and ectopic branches. These disruptions of synaptic connectivity from FGFR misexpression reduced the animal’s behavioral responses, highlighting the importance and sensitivity of the pSc neuron connectivity to proper circuit function, and the sensitivity to FGFR expression in synaptic targeting. In *Drosophila*, both FGFRs *Btl* and *Htl* activate similar pathways with only a small variation in protein affinity [20, 38]. FGFR functional redundancy could explain the similar effects of overexpression and knockdown of *Btl* and *Htl* in our results.

The axonal outgrowth and branching functions of FGF ligands have also been observed in cell culture in the mammalian nervous system [4, 5, 9]. Previous studies have also demonstrated the contributions of FGFR signaling to other targeting molecules, such as *slit1* [39]. In our study, we demonstrate that the axonal branch outgrowth and targeting function of FGFRs is critical for proper synaptic connectivity and circuit function. Furthermore, the stereotyped pSc axonal targeting errors that occurred in *Btl* and *Htl* mutants (Figs. 2f, 3f) suggest that FGFRs are involved in recognizing specific axonal or synaptic targets at distinct branching points or extracellular locations, where loss or gain of FGFR function results in specific misrouting errors.

The axonal outgrowth function of FGFR may be present in all mechanosensory neurons. Using a different Gal4 driver, *DC1.4-Gal4*, which is specific for DorsoCentral mechanosensory neurons, we expressed dsRNA *Btl* (*n* = 6) and observed a loss of some axonal branches (Additional file 1: Figure S2). Overexpression of *Btl* (*n* = 10) resulted in excess branching along the midline. However, the extent to which FGFRs are expressed in other mechanosensory neurons is not known.

FGF signaling has also been implicated in glial migration, differentiation and formation of the neuronal sheath in *Drosophila* [40, 41]. FGFs can control neural circuit formation through epidermal-glial and neuronal-glial interactions to specify glia, and subsequently, synapse location [42]. However, a glial source of FGFs did not seem to influence pSc axonal targeting when the pan-glial driver *repo-Gal4* was used to knock down the FGFs *Bnl* or *Pyr* (Additional file 1**:** Figure S3). These pan-glial RNAi *Bnl* and RNAi *Pyr* animals were very weak and developmentally delayed, making the results difficult to interpret. Thus, the cellular source of FGF ligands and the cellular mechanisms of FGFR activation and signaling in mechanosensory neurons remain unknown. Further examinations of the relationships and expression patterns of the FGF ligands *Bnl*, *Pyr*, and *Ths* and their FGFRs *Btl* and *Htl* within neurons and their target cells will help elucidate the roles of FGF signaling in axonal outgrowth and synaptic targeting.

## Supplementary information

**Additional file 1: Table S1.** Quantitative analysis of axonal arbors for each genotype. **Figure S1.** A schematic of the wildtype pSc axonal arbor was established based on branch size and location. **Figure S2.** *Btl* is required for proper axonal targeting in the pDc mechanosensory neuron. **Figure S3.** FGF ligands and FGFRs genetic analyses in glial cells.

## Data Availability

The datasets, analyses, and materials used in the current study are available from the corresponding author on reasonable request.
