# Supplementary material for: FGF receptors are required for proper axonal branch targeting in Drosophila
Source: Mol Brain. 2019 Oct 24;12:84. doi: 10.1186/s13041-019-0503-y (PMC6814129; doi:10.1186/s13041-019-0503-y)
Supplement: Supplementary file 1 — Additional file 1: Table S1. Quantitative analysis of axonal arbors for each genotype. Figure S1. A schematic of the wildtype pSc axonal arbor was established based on branch size and location. Figure S2. Btl is required for proper axonal targeting in the pDc mechanosensory neuron. Figure S3. FGF ligands and FGFRs genetic analyses in glial cells. [file 13041_2019_503_MOESM1_ESM.pdf]

Table S1 – Quantitative analysis of axonal arbors for each genotype

**Genotype**	***n***	**Number of branches ± S.D.**	**Total Arbor Size ± S.D. (µm)**	**Frequency of midline errors**
Control<br>w[*]; 455-Gal4 / +	19	17.68 ± 3.83	776.3 ± 113.7	5%
Btl null<br>*w[*]; P{w[+mC]=GAL4-btl.S-hsFLP}2, P{w[+mC]=UASp-Act5C.T:GFP}2; btl[724] P{w[+mW.hs]=FRT(w[hs])}2A P{ry[+t7.2]=neoFRT}82B/TM3, P{w[+mC]=tubP-GAL80}3, Sb[1]*	10	14.80 ± 1.135	515.5 ± 59.46	30%
Btl RNAi<br>w[*]; 455-Gal4 / P{y[+t7.7] v[+t1.8]=TRiP.HMS02038}attP2	32	16.35 ± 3.817	650.0 ± 116.0	38%
Btl overexpression<br>w[*]; 455-Gal4 / P{w[+mC]=UAS-btl::GFP-S65T}3	34	24.63 ± 6.81	910.4 ± 180.5	55%
Btl rescue<br>*w[*]/ hsFlp elav-Gal4 UAS-CD8-GFP; P{w[+mC]=UAS-btl::GFP-S65T}3, P{w[+mC]=UASp-Act5C.T:GFP}2; btl[724] P{w[+mW.hs]=FRT(w[hs])}2A P{ry[+t7.2]=neoFRT}82B/TM3, P{w[+mC]=tubP-GAL80}3, Sb[1]*	19	17.17±3.682	887.8±315.2	16%
Htl RNAi<br>w[*]; 455-Gal4 / *P{y[+t7.7] v[+t1.8]=TRiP.HMJ22375}attP40*	38	13.84 ± 3.00	674.9 ± 138.1	26%
Htl DN<br>w[*]; 455-Gal4 / P{w[+mC]=UAS-htl.DN.M}33-B40; P{w[+mC]=UAS-htl.DN.M}33-B61	11	13.91±5.166	662.4 ± 151.4	45%
Htl overexpression<br>w[*]; 455-Gal4 / +; P{w[+mC]=UAS-htl.M}YYDFR-F16 / +	21	21.90 ± 4.182	1100 ± 198.7	57%
Htl rescue<br>w[*]; 455-Gal4 / P{w[+mC]=UAS-htl.DN.M}33-B40; P{w[+mC]=UAS-htl.DN.M}33-B61 / P{w[+mC]=UAS-htl.M}YYDFR-F16	10	14.50±1.581	681.1 ±56.36	20%

Bnl RNAi<br>w[*]; 455-Gal4 / +;; P{y[+t7.7]<br>v[+t1.8]=TRiP.HMS01046}attP2	20	11.73±2.50	704.7 ± 128.7	35%
Pyr RNAi<br>w[*]; 455-Gal4 / P{y[+t7.7]<br>v[+t1.8]=TRiP.HMJ30113}attP40	11	15.70±1.661	699.1 ± 156.2	18%

Supplemental Figures and Legends

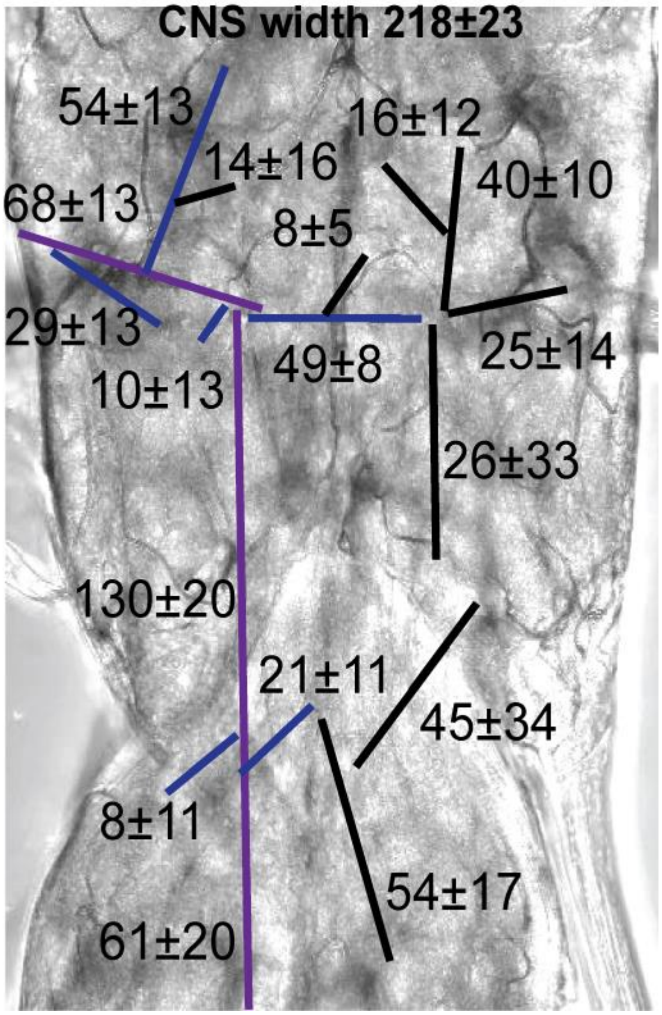

Figure S1 - A schematic of the wildtype pSc axonal arbor was established based on branch size and location. Quantitative analysis of wildtype pSc axonal arbors showed 17 core branches present in over 60% of the control animals. 12 primary and secondary branches were observed in all control animals, while two secondary branches were present in over 80%, and three tertiary branches were found in over 60%. Based on this pSc skeleton, only seven variable branches were detected in less than 10% of control animals. Purple lines represent primary branches, blue lines represent secondary branches, black lines represent tertiary branches. Values are expressed in µm ± S.D.

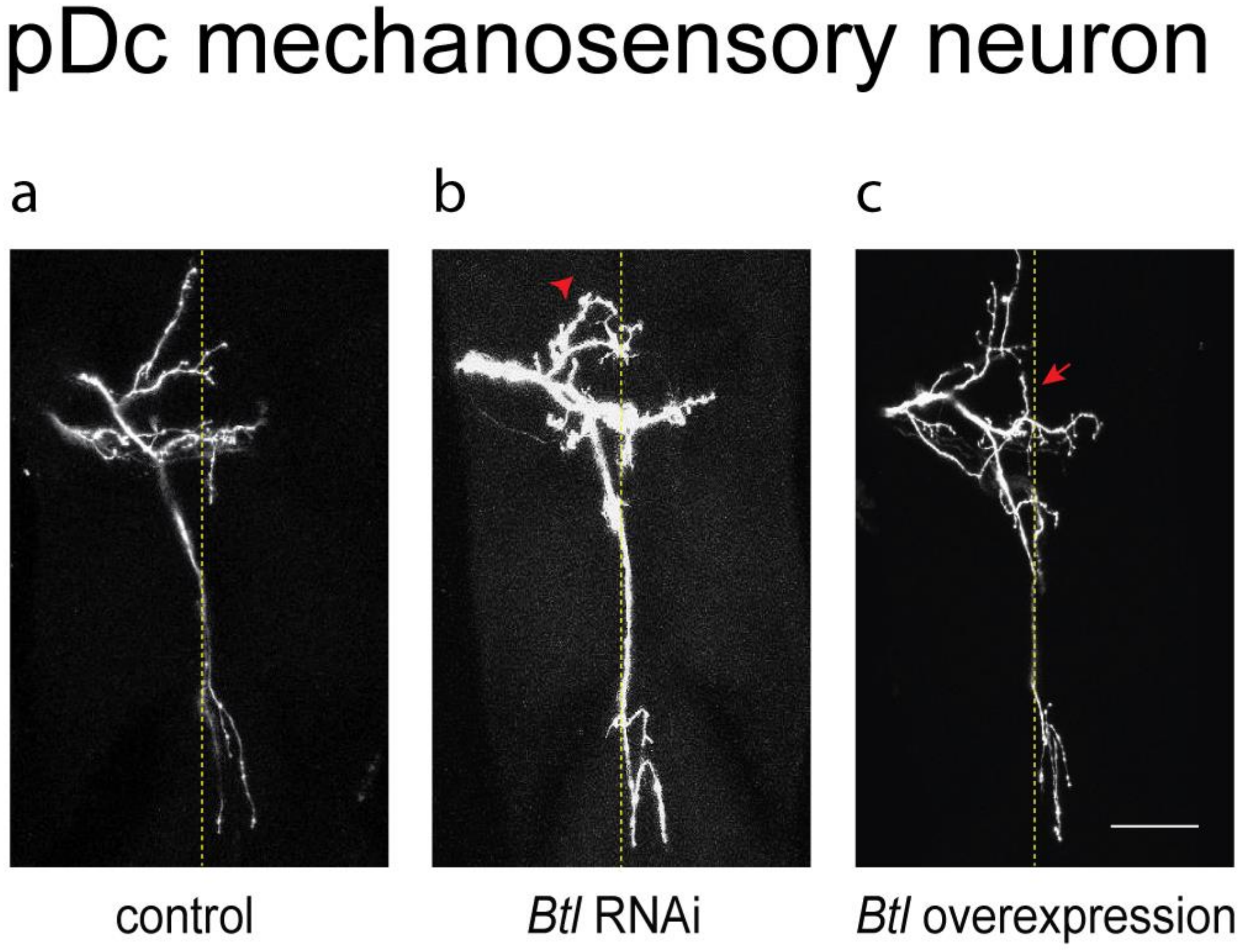

Figure S2 - *Btl* is required for proper axonal targeting in the pDc mechanosensory neuron. (a) The wildtype pDc neuron has a unique stereotyped connectivity pattern. (b) *Btl* RNAi expressed within the pDc neurons had a higher frequency of absent core branches compared to control. (c) *Btl* overexpression had a higher frequency of ectopic branches. Scale bar represents 50μm. Arrowheads point to absent branches, and arrows point to ectopic branches.

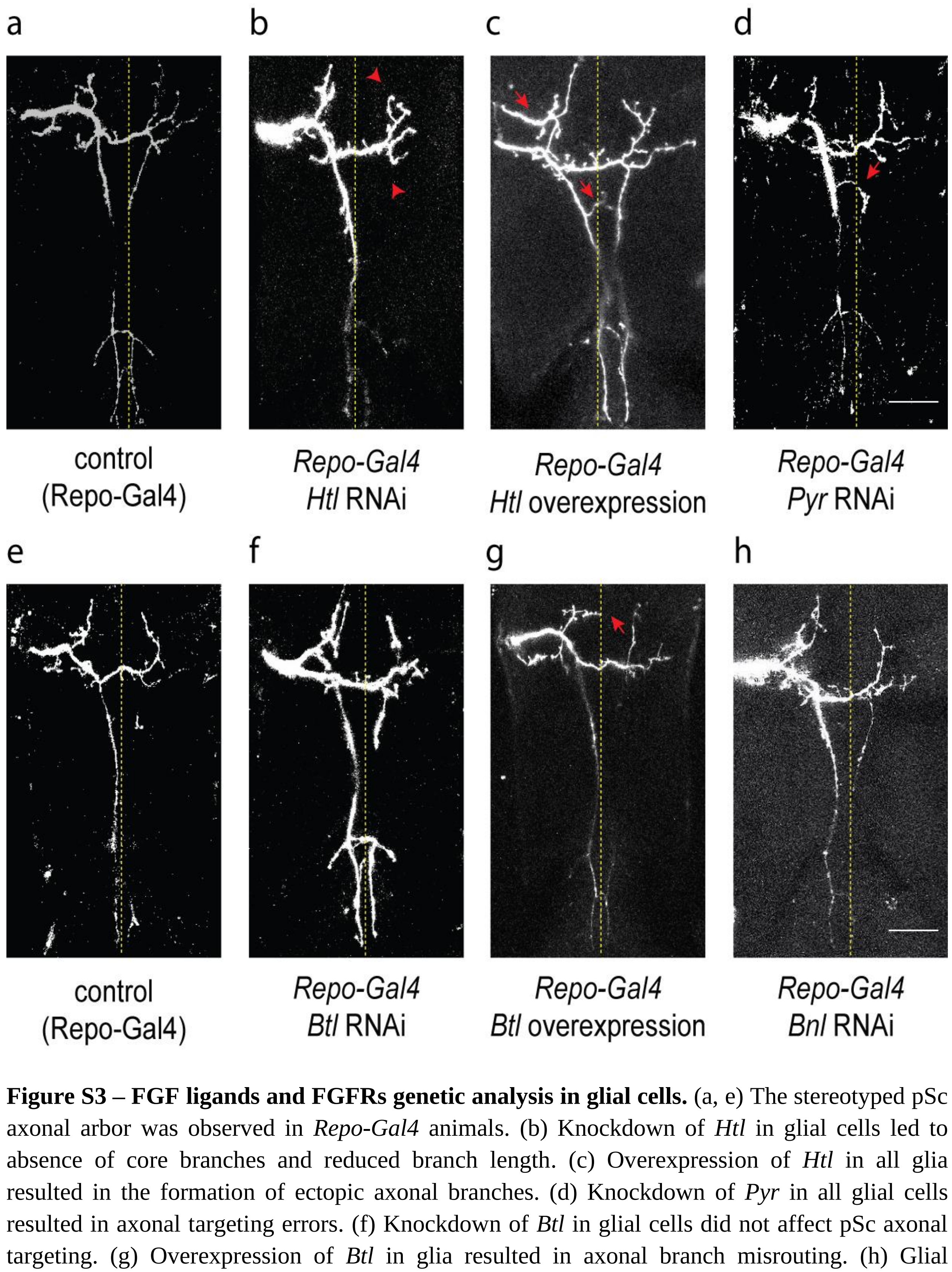

Figure S3 – FGF ligands and FGFRs genetic analysis in glial cells. (a, e) The stereotyped pSc axonal arbor was observed in *Repo-Gal4* animals. (b) Knockdown of *Htl* in glial cells led to absence of core branches and reduced branch length. (c) Overexpression of *Htl* in all glia resulted in the formation of ectopic axonal branches. (d) Knockdown of *Pyr* in all glial cells resulted in axonal targeting errors. (f) Knockdown of *Btl* in glial cells did not affect pSc axonal targeting. (g) Overexpression of *Btl* in glia resulted in axonal branch misrouting. (h) Glial knockdown of *Bnl* did not affect the pSc axonal branching pattern. Scale bar represents 50µm. Arrowheads point to absent branches, and arrows point to ectopic branches.
